# The Prognostic Role of Ribosomal Protein S6 Kinase 1 Pathway in Patients With Solid Tumors: A Meta-Analysis

**DOI:** 10.3389/fonc.2019.00390

**Published:** 2019-05-14

**Authors:** Shuo Zhang, Binwu Hu, Xiao Lv, Songfeng Chen, Weijian Liu, Zengwu Shao

**Affiliations:** ^1^Department of Orthopaedics, Union Hospital, Tongji Medical College, Huazhong University of Science and Technology, Wuhan, China; ^2^Department of Orthopaedic Surgery, The First Affiliated Hospital of Zhengzhou University, Zhengzhou, China

**Keywords:** S6 kinase 1, ribosomal protein S6, solid tumors, prognosis, meta-analysis

## Abstract

**Background:** Recent studies supported the predictive role of ribosomal protein S6 kinase 1 (S6K1), phosphorylated S6K1 (p-S6K1), and phosphorylated ribosomal protein S6 (p-S6) for the outcome of cancer patients. However, inconsistent results were acquired across different researches. To comprehensively and quantitatively elucidate their prognostic significance in solid malignancies, the current meta-analysis was carried out utilizing the results of clinical studies.

**Methods:** We conducted the literature retrieval by searching PubMed, Web of Science, EMBASE, and Cochrane library to identify eligible publications. Data were collected from included articles to calculate pooled overall survival (OS), disease-free survival (DFS), recurrence-free survival (RFS), and progression-free survival (PFS). Hazard ratios (HRs) with 95% confidence intervals (CIs) served as appropriate parameters to assess prognostic significance.

**Results:** Forty-four original studies were included, of which 7 studies were analyzed for S6K1, 24 for p-S6K1, and 16 for p-S6. The overexpression of p-S6K1 was significantly associated with poorer prognosis of solid tumor patients in OS (HR = 1.706, 95%CI: 1.369–2.125, *p* < 0.001), DFS (HR = 1.665, 95%CI: 1.002–2.768, *p* = 0.049). However, prognostic role of p-S6K1 in RFS and PFS was not found. The result also revealed that S6K1 and p-S6 were significantly associated with reduced OS (HR = 1.691, 95%CI: 1.306–2.189, *p* < 0.001; HR = 2.019, 95%CI: 1.775–2.296, *p* < 0.001, respectively).

**Conclusions:** The present meta-analysis demonstrated that elevated expression of S6K1, p-S6K1, or p-S6 might indicate worse prognosis of patients with solid tumors, and supported a promising clinical test to predict solid tumor prognosis based on the level of S6K1 pathway.

## Introduction

In view of high morbidity and mortality all over the world, cancer is well-documented as a global health concern resulting in enormous socioeconomic costs (1–3). Recently, the progression of early diagnosis and the development of various treatments dramatically improved the outcomes of most malignancies (4, 5). However, the prognosis of cancer patients is still unsatisfactory. On the bright side, molecular analysis of cancer tissues greatly augmented the conventional clinical-pathologic paradigm, and then allowed clinical oncology to usher in a new era of molecular medicine (6, 7). Therefore, further researches are in urgent need to explore applicable biomarkers to predict the prognosis of malignancies or work as therapeutic targets.

Dysregulation of the mammalian target of rapamycin (mTOR) pathway components has been reported in various cancers (8–11), and previous meta-analyses have revealed that the mTOR pathway proteins could predict unfavorable cancer prognosis (12, 13). S6K1 is widely accepted as a critical downstream point of mTOR pathway (14). Several hypothetical patterns have been put forward for the regulation of S6K1 phosphorylation, which endows S6K1 with biological function. In a widely accepted model, the mTOR complex 1 (mTORC1) phosphorylates S6K1 hydrophobic motif at Thr389, and the process could be inhibited by rapamycin treatment (14). Other pathways are also supposed to participate in the activation of S6K1, including phosphoinositide 3-kinase (PI3K) signaling (15), mitogen activated protein kinase (MAPK) signaling (16), and even S6K1 autophosphorylation (17). S6K1 plays key roles in diverse cellular processes, including mRNA processing, protein synthesis, cell growth, and homeostasis (18). Accordingly, it was reported that dysregulation of S6K1 was linked to multiple pathologies, especially malignant diseases (14, 19).

Ribosomal protein S6, which serves as a component of 40S ribosomal subunit, is one of the best-characterized kinase effector of p-S6K1 and plays a fundamental role in the control of cell survival and proliferation (14). The phosphorylation of all five sites of S6 (Ser236, Ser235, Ser240, Ser244, and Ser247) could be carried out by S6K1. When phosphorylated by S6 kinases, p-S6 increases the selective translation of a specific subclass of mRNAs with an oligopyrimidines tract at the 5′ untranslated region (5′TOP mRNA) (20). Relevant evidence has been provided for the participation of p-S6 in pancreatic cancer development (21). Besides phosphorylating S6, S6K1 also participates in the regulation of translation initiation and elongation in other ways (22), including activating eukaryotic initiation factor 3 (eIF3) (23), and eukaryotic translation initiation factor 4B (eIF4B) (24).

Previous studies have shown that S6K1, p-S6K1, and p-S6 are abnormally activated in a wide range of cancer types, potentially in relation to prognosis. However, whether S6K1, p-S6K1, and p-S6 could be regarded as prognostic biomarkers and whether the high or low expression of S6K1 pathway is more adverse for the prognosis of solid tumors remains unknown. In addition, to our knowledge, there is no previous comprehensive meta-analysis about the prognostic significance of S6K1 pathway. Therefore, the present meta-analysis was conducted aiming at evaluating the prognostic value of S6K1 pathway in patients with solid tumors.

## Materials and Methods

### Search Strategy

Two researchers (SZ and BH) performed a systematic literature search in PubMed, Web of Science, EMBASE, and Cochrane library to obtain relevant articles up to November 1, 2018. The search query was “S6K1 OR p70S6K OR ribosomal protein S6 kinase OR phosphorylated S6K1 OR p-S6K1 OR phosphorylated p70S6K OR p-p70S6K OR S6 OR ribosomal protein S6” AND “cancer OR tumor” AND “survival OR prognosis.” The reference lists of included studies and relevant review articles were also retrieved for eligible articles.

### Study Selection

The articles were included on condition that they met all the following criteria. (1) Studies must contain the exploration of the association between the expression levels of S6K1, p-S6K1, or p-S6 and overall survival (OS), disease-free survival (DFS), recurrence-free survival (RFS) or progression-free survival (PFS) in any type of solid tumor. (2) The patients had to be divided into high or positive expression group and low or negative expression group according to the expression level of S6K1, p-S6K1, or p-S6. (3) Sufficient data was shown in publications for us to obtain hazard ratios (HRs) and 95% confidence intervals (CIs). (4) In consideration of the heterogeneity of different evaluation and cut-off methods for protein expression, only studies assessing tumor tissues by immunohistochemistry (IHC) staining were included but studies performing western blot (WB), reverse phase protein array (RPPA), enzyme linked immunosorbent assay (ELISA) or polymerase chain reaction (PCR) were excluded in the current meta-analysis.

Studies not in English or not on humans were excluded. For the studies with duplicate data, only the most recent publication was included.

### Data Extraction and Quality Assessment

Two investigators (SZ and BH) reviewed all eligible studies and extracted study characteristics carefully, including author's name, publication year, country, clinicopathological data, phosphorylation site of p-S6K1 or p-S6, study design, treatment information and follow-up duration. In the current analysis, event-free survival (EFS) was defined as a term free of disease, recurrence or progression. We obtained the reported HRs and 95% CIs directly from the publications or from Kaplan-Meier curves by using Engauge Digitizer version 9.8.

The methodological quality of the included researches was assessed by Newcastle-Ottawa Scale (NOS) especially for cohort studies. NOS ranges from 0 to 9, involving selections of exposed cohort, comparability of cohorts and outcome assessment. Researches scoring 6 or more out of 9 were defined as high quality. Rating of studies was carried out by two independent raters (WL and SC) with disagreements resolved by a third rater (BH).

### Data Analysis

Log-transformed HRs with 95% CIs were pooled by Stata software statistical software version 14.0 (Stata Corporation, College Station, TX, USA). The heterogeneity across the studies was measured by Cochran Q test and Higgins I^2^ statistic. Fixed-effect model (Mantel-Haenszel) was performed if heterogeneity was not observed (I^2^ < 50%); otherwise (I^2^ ≥ 50%), random-effect model (DerSimonian and Laird) was considered to be more appropriate. An HR > 1 indicates a poorer prognosis in the patients with biomarker-overexpressed tumors. In addition, we conducted sensitivity analyses to inspect the stability of all summarized outcomes and estimated the potential for publication bias through Begg's test and Egger's linear regression test (25). Galbraith plot was considered appropriate to further explore the sources of heterogeneity and the trim and fill method would be used in the situation of obvious publication bias (26, 27). Due to the limited number of included articles, the subgroup analysis and meta-regression analysis exploring the source of heterogeneity were only conducted in the OS analysis of p-S6K1. All *p*-values were two-sided, and *P* < 0.05 was considered statistically significant.

## Result

### Study Characteristics

**Figure 1:**
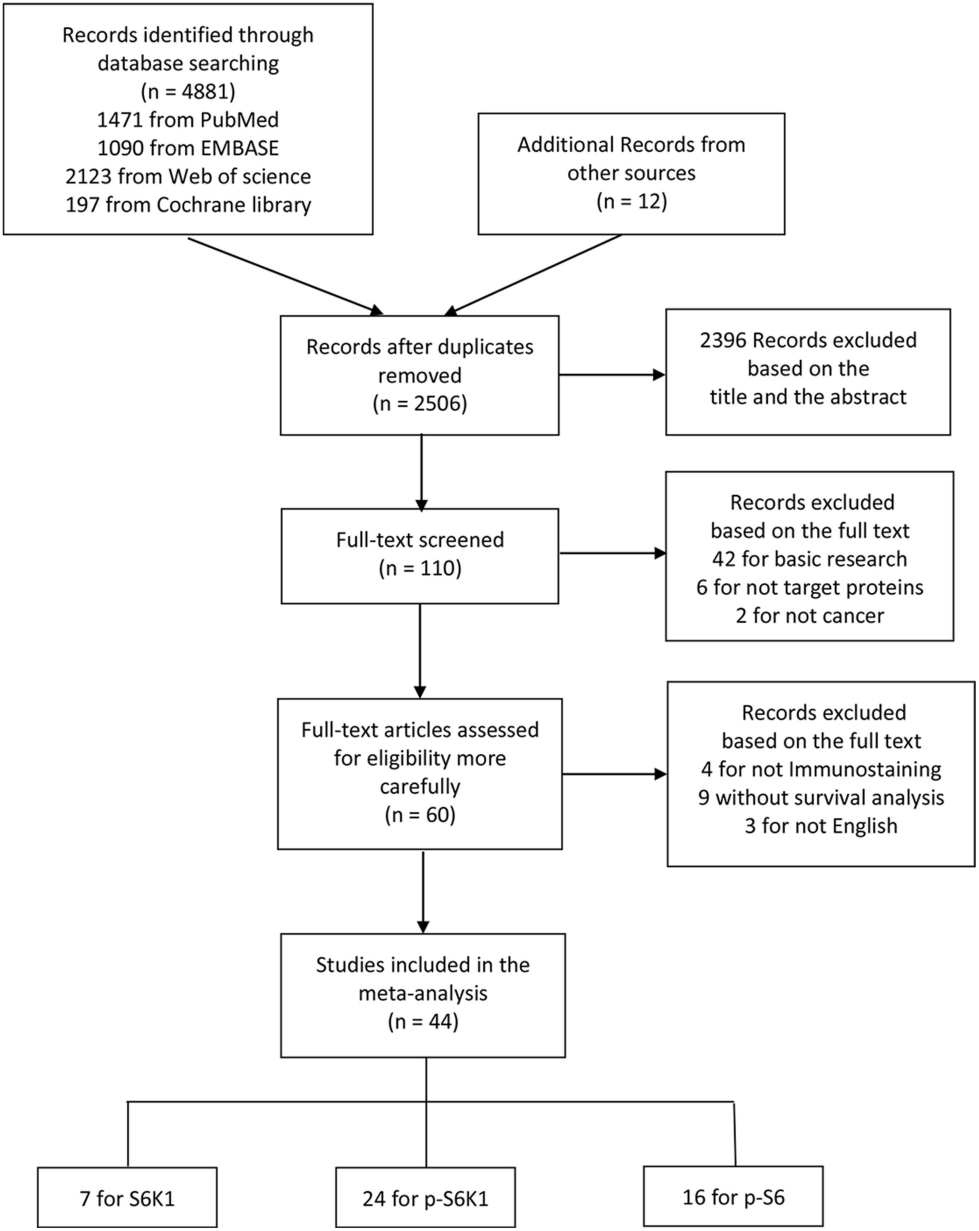
The flow diagram indicating the process of study selection.

Article retrieval flow chart was showed in Figure 1. Among 44 eligible articles (28–71) in this meta-analysis (Table 1), there were 7 for S6K1, 24 for p-S6K1, and 16 for p-S6. The characteristics of included studies are shown in Supplementary Table 1. In summary, (1) the sample size ranges from 30 to 1072; (2) the year of publication ranges from 2004 to 2018; (3) the follow-up duration ranges from 25 to 291 months; (4) 18 of these studies were conducted in western countries, while 25 in Asia and 1 in Africa; (5) HRs with 95%CIs were obtained directly from all but six included publications; (6) well-defined cut-off values were stated in each included study. Histoscore (H-score) according to staining intensity and positive proportion by IHC was widely applied.

**Table 1:** Included studies.

**Target protein**	**OS**		**EFS**		
	**M**	**Not M**	**DFS**	**PFS**	**RFS**
S6K1	(33, 39, 59)	(28, 35, 71)	(28, 33)	(71)	(51)
p-S6K1	(34–36, 38, 41, 50, 55, 59, 65, 67, 68, 70)	(30, 31, 46, 54, 60, 71)	(36, 55, 56)	(49, 58, 60, 71)	(47, 48, 54, 57)
p-S6	(32, 37, 62, 64, 70)	(42, 44, 45, 52, 61, 63)	(29, 44, 66)	(40, 43, 62)	(43, 53, 63, 69)

*M, multivariate analysis*.

### Prognostic Value of p-S6K1 in Solid Tumors

#### Overall Survival (OS)

**Figure 2:**
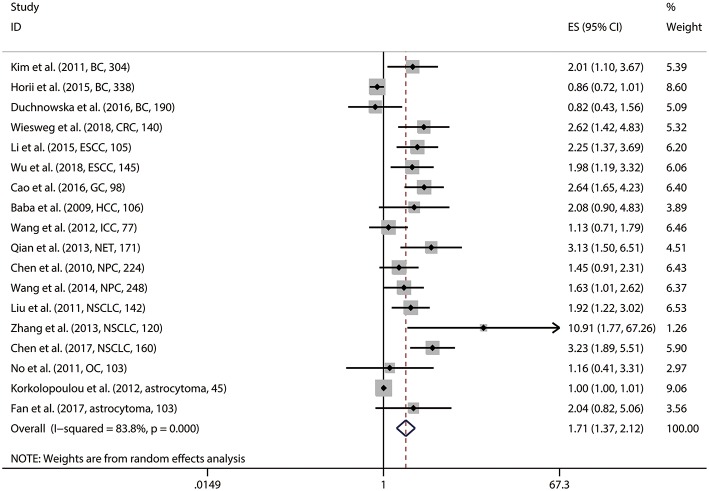
Meta-analysis of the pooled HRs of OS for patients with abnormally expressed p-S6K1. In the forest plots, each study ID was set as the following format: authors (year, tumor type, and sample size).

**Figure 3:**
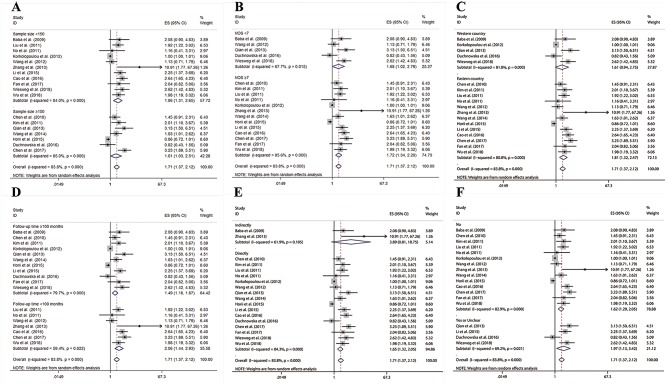
Results of subgroup analysis of pooled HRs of OS for cancer patients. **(A)** Subgroup analysis stratified by sample size. **(B)** Subgroup analysis stratified by NOS score. **(C)** Subgroup analysis stratified by region. **(D)** Subgroup analysis stratified by follow-up period. **(E)** Subgroup analysis stratified by source of HRs. **(F)** Subgroup analysis stratified by preoperative treatment.

Pooled analyses of 18 studies involving 2,819 patients showed that p-S6K1 overexpression was significantly associated with worse OS (HR = 1.706, 95%CI: 1.369–2.125, *p* < 0.001) (Table 2; Figure 2). Significant inter-study heterogeneity (Cochrane Q, *p* < 0.001; I^2^ = 83.8%) asked for a random-effect model. We further conducted subgroup analyses and meta-regression analysis to explore the source of heterogeneity by factors of sample size (≥150 and <150), NOS score (<7 and ≥7), region (western country and eastern country), follow-up period (≥100 months and <100 months), source of HRs (HRs obtained directly and indirectly) and preoperative treatment (no and yes or unclear). Two subgroup factors altered the significant relationship between p-S6K1 expression and OS in the six factors above (Table 3) (Figure 3). However, meta-regression analysis indicated that these six factors were not the source of heterogeneity.

**Table 2:** Pooled HRs, heterogeneity and publication bias for OS, DFS, PFS, RFS, and EFS in cancer patients with abnormal expression level of S6K1, p-S6K1, and p-S6.

**Factor**	**No. of studies**	**Model**	**Pooled HR (95%CI)**	***P***	**Heterogeneity**		**Publication bias**	
					**I^2^**	***P***	***P* of Begg's test**	***P* of Egger's test**
**S6K1**								
OS	6	F	1.69 (1.31, 2.19)	< 0.001^*^	20.9%	0.276	0.133	0.135
OS from M	3	R	1.99 (0.82, 4.85)	0.128	77.8%	0.011	–	–
EFS	4	F	2.07 (1.49, 2.89)	< 0.001^*^	0.00%	0.484	–	–
**p-S6K1**								
OS	18	R	1.71 (1.37, 2.12)	< 0.001^*^	83.8%	< 0.001	0.596	< 0.001
OS from M	12	R	1.95 (1.36, 2.80)	< 0.001^*^	86.8%	< 0.001	–	–
OS for ESCC	2	F	2.12 (1.48, 3.02)	< 0.001^*^	0.0%	0.732	–	–
OS for NSCLC	3	R	4.52 (1.52, 13.45)	0.007^*^	88.3%	< 0.001	–	–
OS for NPC	2	F	1.53 (1.10, 2.14)	0.012^*^	0.0%	0.725	–	–
OS for BC	3	R	1.08 (0.65, 1.80)	0.766	72.1%	0.028	–	–
DFS	3	R	1.67 (1.00, 2.77)	0.049^*^	70.5%	0.034	–	–
PFS	4	R	1.47 (0.60, 3.63)	0.402	84.8%	0.001	–	–
RFS	4	R	0.72 (0.31, 1.69)	0.454	86.0%	< 0.001	–	–
**p-S6**								
OS	11	F	2.02 (1.78, 2.30)	< 0.001^*^	17.7%	0.275	0.592	0.340
OS from M	5	F	1.78 (1.49, 2.11)	< 0.001^*^	23.0%	0.268	–	–
DFS	3	R	1.54 (0.70, 3.41)	0.287	76.5%	0.014	–	–
PFS	3	F	2.09 (1.10, 3.98)	0.024^*^	0.0%	0.743	–	–
RFS	4	F	2.21 (1.52, 3.23)	< 0.001^*^	46.9%	0.130	–	–

*M, multivariate analysis; R, random-model; F, fixed-model*.

* *p < 0.05*.

**Table 3:** Subgroup analysis of pooled HRs for OS in cancer patients with abnormal expression level of p-S6K1.

**Subgroup analysis**	**No. of cohorts**	**Pooled HR (95% CI)**	**P**	**Heterogeneity**		**P (meta regression)**
				**I^2^**	**P**	
**Sample size**						0.775
<150	11	1.86 (1.31, 2.65)	0.001^*^	84.0%	< 0.001	–
≥150	7	1.61 (1.03, 2.51)	0.037^*^	85.0%	< 0.001	–
**NOS score**						0.718
<7	5	1.68 (1.02, 2.79)	0.044^*^	67.7%	0.015	–
≥7	13	1.72 (1.34, 2.20)	< 0.001^*^	85.6%	< 0.001	–
**Region**						0.966
Western country	5	1.61 (0.94, 2.75)	0.082	81.8%	< 0.001	–
Eastern country	13	1.81 (1.32, 2.47)	0.001^*^	80.8%	< 0.001	–
**Follow-up period**						0.378
≥100 months	11	1.49 (1.18, 1.87)	0.001^*^	79.7%	< 0.001	–
<100 months	7	2.06 (1.44, 2.93)	0.002^*^	59.4%	0.022	–
**Source of HRs**						0.182
Directly	16	1.65 (1.32, 2.05)	< 0.001^*^	84.3%	< 0.001	–
Indirectly	2	3.89 (0.81, 18.75)	0.091	61.9%	0.105	–
**Preoperative treatment**						0.543
No	14	1.62 (1.29, 2.05)	< 0.001^*^	82.9%	< 0.001	–
Yes or unclear	4	1.97 (1.13, 3.42)	0.017^*^	69.2%	0.021	–

* *p < 0.05*.

Furthermore, pooled HR of 12 researches performing Cox multivariate analyses revealed high p-S6K1 expression could serve as an independent prognostic predictor for reduced OS of patients with solid malignances (HR = 1.951, 95%CI: 1.357–2.804, *p* < 0.001) (Table 2; Supplementary Figure 1).

We further evaluated the prognostic value of p-S6K1 in certain types of cancer (Table 2). It was found that p-S6K1 predicted poor prognosis of esophageal squamous cell carcinoma (ESCC) (HR = 2.116, 95%CI: 1.481–3.022, *p* < 0.001) (Supplementary Figure 2A), non-small cell lung cancer (NSCLC) (HR = 4.515, 95%CI: 1.516–13.450, *p* = 0.007) (Supplementary Figure 2B) and nasopharyngeal carcinoma (NPC) (HR = 1.535, 95%CI: 1.100–2.141, *p* = 0.012) (Supplementary Figure 2C), but not breast cancer (BC) (HR = 1.081, 95%CI: 0.649–1.801, *p* = 0.766) (Supplementary Figure 2D).

#### Disease-Free Survival (DFS), Recurrence-Free Survival (RFS), and Progression-Free Survival (PFS)

Further, the impact of elevated expression of p-S6K1 on the prognosis of solid tumors was explored in 3 studies with 493 cases for DFS, 4 studies with 425 cases for PFS, and 4 studies with 557 cases for RFS, respectively. Random effect model was suitable for the analyses of DFS (Cochrane Q, *p* = 0.034; I^2^ = 70.5%), PFS (Cochrane Q, *p* = 0.001; I^2^ = 84.8%) and RFS (Cochrane Q, *p* < 0.001; I^2^ = 86.0%) owing to obvious heterogeneity. The consequence displayed that p-S6K1 was significantly associated with reduced DFS (HR = 1.665, 95%CI: 1.002–2.768, *p* = 0.049) (Supplementary Figure 3A), but not PFS (HR = 1.472, 95%CI: 0.596–3.632, *p* = 0.402) (Supplementary Figure 3B) and RFS (HR = 0.722, 95%CI: 0.308–1.693, *p* = 0.454) (Supplementary Figure 3C) in patients with solid malignancies (Table 2).

#### Sensitivity Analysis and Publication Bias

**Figure 4:**
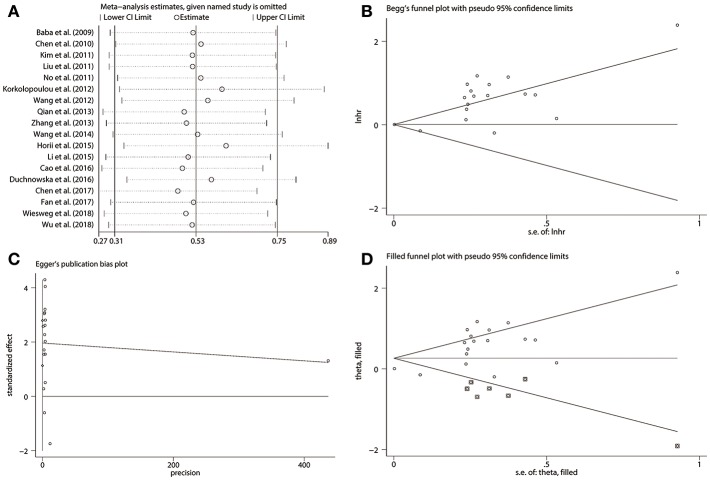
The credibility and stability of OS analysis for p-S6K1 in solid tumors based on 18 researches. Sensitivity analysis plot **(A)** showed the pooled HRs with 95%CIs after omitting any of the studies. The elimination of any studies did not alter the statistical significance. Begg's test **(B)** did not indicate the existence of publication bias (*p* = 0.596). However, significant publication bias was detected by Egger's linear regression test **(C)** (*p* < 0.001). Trim and fill analysis **(D)** additionally filled seven missing studies to adjust the publication bias. Empty circles were original data and empty circles in squares were imputed filled values.

For the analysis of OS, we evaluated the effect of a certain study on the summarized outcomes through sensitivity analysis. The results showed that the elimination of any research did not alter the original statistical significance, further confirming the stability and credibility of the eventual results (Figure 4A). In addition, publication bias in the analysis of the association between p-S6K1 expression and OS was estimated by Begg's test and Egger's linear regression test. Publication bias was not detected by Begg's test (*p* = 0.596) (Figure 4B). Inversely, Egger's linear regression test proved that there was significant publication bias (*p* < 0.001) (Figure 4C). To further explore the impact of potential publication bias on pooled results, we conducted trim and fill analysis, which is a funnel-plot-based method for testing and adjusting the publication bias in meta-analysis. Trim and fill analysis demonstrated that seven studies investigating the relationship between p-S6K1 and OS of solid tumors were potentially unpublished or unavailable (Figure 4D). Moreover, filled result (HR = 1.300, 95% CI: 1.077–1.568, *p* = 0.006) sustained the statistical significance.

### Prognostic Value of S6K1 in Solid Tumors

**Figure 5:**
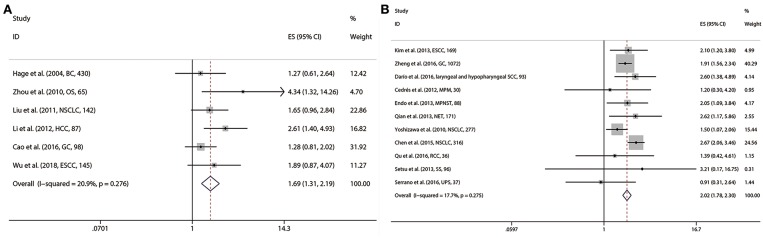
Meta-analysis of the pooled HRs of OS for patients with abnormally expressed S6K1 **(A)** and p-S6 **(B)**.

Pooled analyses were conducted to estimate the prognostic value of S6K1 on six researches comprising 967 patients for OS and four researches involving 1,059 cases for EFS, respectively. Fixed effect model was preferred for the analysis of OS (Cochrane Q, *p* = 0.276; I^2^ = 20.9%) and EFS (Cochrane Q, *p* = 0.484; I^2^ = 0.0%). The results revealed S6K1 overexpression to be significantly associated with worse OS (HR = 1.691, 95%CI: 1.306–2.189, *p* < 0.001) (Figure 5A) and EFS (HR = 2.074, 95%CI: 1.488–2.890, *p* < 0.001) in patients with solid tumors (Table 2).

### Prognostic Value of p-S6 in Solid Tumors

The prognostic value of p-S6 was reported in sixteen observational studies, of which 11 were analyzed for OS, 3 for DFS,3 for PFS, and 4 for RFS. P-S6 was found significantly associated with worse OS (HR = 2.019, 95%CI: 1.775–2.296, *p* < 0.001) (Figure 5B), PFS (HR = 2.092, 95%CI: 1.100–3.979, *p* = 0.024) (Supplementary Figure 4B) and RFS (HR = 2.214, 95%CI: 1.518–3.229, *p* < 0.001) (Supplementary Figure 4C). Statistical significance of p-S6 in DFS was not found in the current meta-analysis (HR = 1.540, 95%CI: 0.696–3.409, *p* = 0.287) (Table 2; Supplementary Figure 4A).

## Discussion

S6K1 pathway was widely reported to be dysregulated in various solid malignancies, and S6K1, p-S6K1, and p-S6 were considered as potential prognostic biomarkers in cancer patients (14, 18). However, negative or even opposite results were also acquired (72). Here, the purpose of this meta-analysis was to provide a reliable and comprehensive summary of the prognostic value of S6K1 pathway dysfunction in solid tumors and to explore its clinical significance.

Through the systematic analysis of 44 independent clinical studies, we found that elevated expression levels of S6K1, p-S6K1, or p-S6 were significantly related to poorer OS in patients with solid tumors. In the subgroup analyses of prognostic value of p-S6K1, two of all six subgroup factors altered the statistical significance. However, the sources of heterogeneity were not ascertained by meta-regression analysis. Furthermore, by pooling HRs from multivariate analysis, we found that p-S6K1 overexpression could act as an independent risk factor for OS in patients with solid tumors. The prognostic value of p-S6K1 in four certain types of tumor was further evaluated. Our results revealed that the prognostic significance of p-S6K1 in OS existed in ESCC, NSCLC and NPC, but not in BC. In addition, our results indicated statistical significance existed in the association between p-S6K1 overexpression and shorter DFS, S6K1 overexpression and shorter EFS, p-S6 overexpression and shorter PFS, p-S6 overexpression and shorter RFS, respectively. However, discrepant results of these analyses might derive from limited number of eligible studies. Therefore, larger-scale, multicenter studies including all stage patients are highly required for more comprehensive analysis in the future.

Obvious heterogeneity was detected in the analysis of the prognostic value of p-S6K1 in OS of solid tumor patients, which would downgrade the evidence potentially. We speculated that discrepant tumor stage and therapeutic regimen might be the sources of heterogeneity. After excluding two studies Korkolopoulou et al. (38) and Horii et al. (54), which were indicated to be the potential sources of heterogeneity by Galbraith plot, I^2^ declined dramatically to 43.6%. Korkolopoulou et al. (38) is the only study exploring p-S6K1 phosphorylated at Thr421/Ser424. In addition, inclusion of breast cancer might cause a part of heterogeneity. Results of the survival analysis of p-S6K1 in OS of breast cancer were inconsistent across different researches and prognostic significance was not obtained in the current studies. Two researches Horii et al. (54) and Duchnowska et al. (60) reported that the expression of p-S6K1 predicted favorable OS without statistical significance. Kim et al. (34) reported that p-S6K1 was an independent prognostic factor in hormone receptor (HR)-positive, but not HR-negative, breast cancer. Furthermore, S6K/p-S6 could exert a pro-tumor effect via regulating the phosphorylation status of ER (73) and the production of vascular endothelial growth factor (VEGF) (74) of breast cancer cells. Moreover, the role of p-S6K1 varied considerably in different specific targeted therapies of breast cancer (34, 47, 75).

Publication bias might exist in the current meta-analysis. The bias might derive from the exclusion of non-English articles and the retrieval method limited to studies published in peer-reviewed journals. A majority of the included studies tended to report positive results so the prognostic value of S6K1, p-S6K1, or p-S6 might be overestimated to some extent. In addition, we speculated that inconsistent calculation methods and different sensitivities resulted in the discrepant statistical significance between Begg's test and Egger's test. In view of the issue of publication bias, we conducted trim and fill analysis and acquired a statistically significant result again.

The mechanisms that S6K1 pathway influences the prognosis of solid tumor patients have been thoroughly elucidated. S6K1 plays a critical role in cell metabolism and growth, potentially in association with malignant biological properties, including cancer cell growth, proliferation and migration. It was reported that S6K1-mediated phosphatidylinositol 4-phosphate 5-kinase type I γ (PIPKIγ90) phosphorylation regulated the development of cancer cell migration and invasion (76). Another study demonstrated that the mTOR/S6K1 was hyperactivated in breast cancer cells, and inhibition of S6K1 could downregulate p-S6 and impede cell growth and migration (77, 78). Similarly, S6K1 was found to stimulate ovarian cancer cell invasion by activating matrix metalloproteinase (MMP)-9 (79). Amaral et al. (80) revealed that S6K1 overexpression strengthened prostate cancer cell viability, migration, and tumor formation *in vivo*. Previous research also indicated that p-S6 attenuated p53-mediated tumor suppression in pancreatic cancer and positively related to pancreatic intraepithelial neoplasia (PanIN) (21).

However, a few limitations of the current meta-analysis should be underlined. First, there is a considerable discrepancy of the cut-off methodologies of target protein expression among included studies, although H-score according to staining intensity and positive proportion was widely used. Second, HRs or CIs of six studies could not be directly extracted from original publications. Errors possibly existed in the process of calculating HRs and 95%CIs by Kaplan-Meier curves. Third, included tumor types varied dramatically. Their pathological pattern, degree, clinical stage and therapeutic regimen differed from each other. Fourth, in order to achieve internal consistency of the evaluation methodology, only studies estimating protein expression by IHC staining were included in the current meta-analysis. Fifth, the outcomes of the current meta-analysis might be limited by the retrospective nature of the majority of included studies.

## Conclusions

Our study demonstrated that S6K1, p-S6K1, and p-S6 might be unfavorable prognostic biomarkers in patients with solid tumors. However, the value of S6K1 pathway as anti-cancer targets is still unclear. Therefore, more prospective, high-quality and multi-center clinical trials are urgently needed to explore the impact of potent inhibitors targeting S6K1 pathway on the prognosis of solid tumor patients.

## Author Contributions

SZ and BH collected, extracted, and analyzed the data. WL and SC performed quality assessment and analyzed the data. XL wrote the paper. ZS conceived and designed this study. All authors read and approved the final manuscript.

### Conflict of Interest Statement

The authors declare that the research was conducted in the absence of any commercial or financial relationships that could be construed as a potential conflict of interest.

## Abbreviations

ESCC: esophageal squamous cell carcinoma
GC: gastric cancer
CRC: colorectal cancer
HCC: hepatocellular carcinoma
ICC: intrahepatic cholangiocarcinoma
NPC: nasopharyngeal carcinoma
NSCLC: non-small cell lung cancer
RCC: renal cell carcinoma
BUC: bladder urothelial carcinoma
BC: breast cancer
OC: ovarian cancer
OS: osteosarcoma
SS: synovial sarcoma
UPS: undifferentiated pleomorphic sarcoma
MPNST: malignant peripheral nerve sheath tumor
MPM: malignant pleural mesothelioma
SCC: squamous cell carcinoma
NET: neuroendocrine tumor
FIGO: International Federation of Gynecology and Obstetrics
UICC: Union for International Cancer Control
AJCC: American Joint Committee on Cancer
OS: overall survival
DFS: disease-free survival
RFS: recurrence-free survival
PFS: progression-free survival
EFS: event-free survival
ROC: receiver-operating characteristic
IRS: immunoreactive score
NOS: Newcastle-Ottawa Scale
NS: not stated
U: univariate analysis
M: multivariate analysis
H-score: Histoscore according to IHC.
