# Supplementary material for: The Prognostic Role of Ribosomal Protein S6 Kinase 1 Pathway in Patients With Solid Tumors: A Meta-Analysis
Source: Front Oncol. 2019 May 14;9:390. doi: 10.3389/fonc.2019.00390 (PMC6527894; doi:10.3389/fonc.2019.00390)
Supplement: Supplementary file 1 [file Table_1.DOC]

**Supplementary Table. 1 Characteristics of included** studies

| **Authors** | **Year** | **Region** | **Tumor type** | **Stage or Grade** | **Sample size** | **biomarker** | [**Phosphorylation site**](../../../../C:/Users/lenovo/AppData/Local/youdao/dict/Application/7.2.0.0703/resultui/dict/javascript:%3B) | **Endpoints** | **Available Survival**  **analysis** | **Cut-off value** | **Preoperative treatment** | **Follow-up period** | **NOS scores** | **Data obtainment^a^** |
| --- | --- | --- | --- | --- | --- | --- | --- | --- | --- | --- | --- | --- | --- | --- |
| **Hage et al.** | **2004** | Netherlands | BC | early-stage | 430 | S6K1 | — | OS, DFS | U, M | staining score difference between tumor normal epithelial cells ≥2 | Unclear | 130m | 6 | 1 |
| **Campbell et al.** | **2008** | The U.K. | RCC | NS | 107 | p-S6 | Ser235/236 | DFS | No | According to the most informative split | No | 142m | 7 | 2 |
| **Baba et al.** | **2009** | Germany | HCC | NS | 106 | p-S6K1 | NS | OS | No | Positive: staining extent >50% | No | 100m | 6 | 2 |
| **Chen et al.** | **2010** | China | NPC | I-IV | 224 | p-S6K1 | Thr389 | OS | U | Positive: IRS ≥4 | No | 200m | 7 | 1 |
| **Yoshizawa et al.** | **2010** | USA | NSCLC | I-IV | 277 | p-S6 | NS | OS | U, M | Positive: total score ≥3 | No | 163m | 7 | 1 |
| **Zhou et al.** | **2010** | China | OS | II-III | 65 | S6K1 | — | OS, DFS | M | Positive: staining of >70% of cells | Yes | 60m | 7 | 1 |
| **Kim et al.** | **2011** | Korea | BC | NS | 304 | p-S6K1 | NS | OS | M | Positive: score >0 | No | 130m | 7 | 1 |
| **Liu et al.** | **2011** | China | NSCLC | I-IV | 142 | p-S6K1 | Ser424 | OS | U, M | Positive: total score =2–9 | No | 87m | 7 | 1 |
|  |  |  |  |  |  | S6K1 | — | OS | U | Positive: total score =2–9 |  |  |  | 1 |
| **No et al.** | **2011** | Korea | OC | FIGO I–IV | 103 | p-S6K1 | Thr389 | OS, DFS | M | Positive: stained cells >1% | No | 82m | 7 | 1 |
| **Cedrés et al.** | **2012** | Spain | MPM | NS | 30 | p-S6 | NS | OS, PFS | M | Median H‑score | Unclear | 40m | 6 | 1 |
| **Korkolopoulou et al.** | **2012** | Greece | astrocytoma | NS | 45 | p-S6K1 | Thr421/Ser424 | OS | M | ROC curve | No | 104m | 7 | 1 |
| **Li et al.** | **2012** | China | HCC | NS | 87 | S6K1 | — | OS | U, M | Positive: score = 4–9 | No | 80m | 7 | 1 |
| **Mueller et al.** | **2012** | USA | gliomas | Grade I-IV | 48 | p-S6 | Ser240/244 | PFS | No | Positive: score ≥2 | Unclear | 210m | 6 | 2 |
| **Wang et al.** | **2012** | China | ICC | NS | 77 | p-S6K1 | Thr389 | OS | U, M | Median H‑score | No | 50m | 6 | 1 |
| **Endo et al.** | **2013** | Japan | MPNST | AJCC I-IV | 88 | p-S6 | Ser235/236 | OS | U | Positive: staining intensity: tumor cells ≥ endothelial cells | No | 291m | 7 | 1 |
| **Fahmy et al.** | **2013** | Canada | BUC | AJCC pTaHG- pT1 | 142 | p-S6 | Ser235/236 | PFS, RFS | M | Median tumor H‑score | Unclear | 118m | 6 | 1 |
| **Kim et al.** | **2013** | Korea | ESCC | I-IV | 169 | p-S6 | Ser240/244 | OS, DFS | U | Positive: score >4 | Unclear | 100m | 6 | 1 |
| **Qian et al.** | **2013** | USA | NET | I-IV | 171 | p-S6K1 | Thr389 | OS | U, M | Positive: IRS ≥6 | Unclear | 108m | 6 | 1 |
|  |  |  |  |  |  | p-S6 | Ser240/244 | OS | U, M | Positive: IRS ≥4 |  |  |  |  |
| **Setsu et al.** | **2013** | Japan | SS | AJCC II-IV | 96 | p-S6 | Ser235/236 | OS | No | Positive: >10% tumor cells stained more strongly than adjacent endothelial cells | No | 278m | 6 | 2 |
| **Zhang et al.** | **2013** | China | NSCLC | I-IV | 120 | p-S6K1 | NS | OS | No | Positive: score =2-9 | No | 60m | 7 | 2 |
| **Beelen et al.** | **2014** | Netherlands | BC | I-III | 95 | p-S6K1 | NS | RFS | U | Median H‑score | Unclear | 150m | 6 | 1 |
| **Kim et al.** | **2014** | Korea | BUC | NS | 95 | p-S6K1 | Ser371 | RFS | U, M | Median H‑score (93) | Unclear | 120m | 6 | 1 |
| **Nishikawa et al.** | **2014** | Japan | RCC | NS | 48 | p-S6K1 | NS | PFS | U | Positive: staining score =1, 2 | No | 25m | 6 | 1 |
| **Wang et al.** | **2014** | China | NPC | I-IV | 248 | p-S6K1 | Thr389 | OS | M | Positive: total score =2–12 | No | 120m | 8 | 1 |
| **Bostner et al.** | **2015** | Sweden | BC | NS | 418 | S6K1 | — | RFS | U | Positive: strong intensity | Unclear | 300m | 6 | 1 |
| **Chen et al.** | **2015** | China | NSCLC | I-IV | 316 | p-S6 | Ser235/236 | OS | U | Positive: score =3–9 | No | 60m | 7 | 1 |
| **Haddad et al.** | **2015** | USA, Germany, France, Italy | RCC | NS | 529 | p-S6 | Ser235/236 | RFS | U | Positive: H-score >2 | Unclear | 220m | 6 | 1 |
| **Horii et al.** | **2015** | Japan | BC | I-III | 338 | p-S6K1 | Thr389 | OS, RFS | U | According to staining intensity and positive proportion | No | 185m | 7 | 1 |
| **Li et al.** | **2015** | China | ESCC | AJCC I-IV | 105 | p-S6K1 | Thr389 | OS, DFS | M | Positive: staining score=1,2 | No | 146m | 8 | 1 |
| **Ma et al.** | **2015** | China | BC | I-IV | 285 | p-S6K1 | Thr389 | DFS | M | Positive: IRS >3 | No | 80m | 7 | 1 |
| **Nishikawa et al** | **2015** | Japan | BUC | NS | 49 | p-S6K1 | NS | RFS | U | Positive: staining score =1,2 | No | 70m | 6 | 1 |
| **Azim et al.** | **2016** | Egypt | BC | NS | 33 | p-S6 | NS | RFS | No | Positive: staining score ≥2 | Unclear | 60m | 6 | 2 |
| **Benslama et al.** | **2016** | France | NET | NS | 42 | p-S6K1 | NS | PFS | M | Positive: tumor cells expression level > internal controls | Unclear | 60m | 6 | 1 |
| **Cao et al.** | **2016** | China | GC | I-IV | 98 | p-S6K1 | NS | OS | U, M | Positive: staining score >2 | No | 70m | 7 | 1 |
|  |  |  |  |  |  | S6K1 | — | OS | U, M | Positive: staining score >2 |  |  |  | 1 |
| **Darío et al.** | **2016** | Spain | laryngeal and hypopharyngeal SCC | I-IV | 93 | p-S6 | Ser235/236, Ser240/244 | OS | U | Positive: stained cells >10% | No | 95m | 7 | 1 |
| **Duchnowska et al.** | **2016** | Poland | BC | I-IV | 190 | p-S6K1 | NS | OS, PFS | U, M | Maximize HR between two subpopulations | Yes | 242m | 6 | 1 |
| **Qu et al.** | **2016** | China | RCC | NS | 36 | p-S6 | Thr389/412 | OS. PFS | U, M | Positive: tumors with moderate intensity (++) >66% or strong intensity (+++) >33% | No | 87m | 7 | 1 |
| **Serrano et al.** | **2016** | Spain | UPS | AJCC II-III | 37 | p-S6 | Ser240/244 | OS, RFS | U | Analyze variables distributions by density plots and choose curve points after asymmetrical distributions | No | 62m | 6 | 1 |
| **Zheng et al.** | **2016** | China | GC | I-IV | 1072 | p-S6 | Ser235/236 | OS | M | According to staining intensity and positive proportion | No | 60m | 7 | 1 |
| **Chen et al.** | **2017** | China | NSCLC | I-IV | 160 | p-S6K1 | Thr389 | OS | U, M | Positive: staining score =3-9 | No | 60m | 8 | 1 |
| **Juan et al.** | **2017** | Spain | oral SCC | I-IV | 125 | p-S6 | Ser235/236, Ser240/244 | DFS | U | Positive: stained cells >10% | No | 60m | 7 | 1 |
| **Fan et al.** | **2017** | China | astrocytoma | I-IV | 103 | p-S6K1 | Thr389 | OS | M | Positive: staining score =4-7 | No | 120m | 8 | 1 |
| **Wiesweg et al.** | **2018** | Germany | CRC | UICC I-IV | 140 | p-S6K1 | Thr389 | OS | M | Positive: IRS ≥4 | Unclear | 120m | 6 | 1 |
| **Wu et al.** | **2018** | China | ESCC | AJCC I-IV | 145 | p-S6K1 | Thr389 | OS, PFS | U | Positive: staining score ≥4 | No | 60m | 7 | 1 |
|  |  |  |  |  |  | S6K1 | — | OS, PFS | U | Positive: staining score ≥4 |  |  |  |  |

a: 1 denoted as obtaining HRs and 95%CIs directly from publications; 2 denoted as HRs calculated from the total number of events, corresponding p value and data from Kaplan-Meier curves.
