# Supplementary material for: The Prognostic Role of Ribosomal Protein S6 Kinase 1 Pathway in Patients With Solid Tumors: A Meta-Analysis
Source: Front Oncol. 2019 May 14;9:390. doi: 10.3389/fonc.2019.00390 (PMC6527894; doi:10.3389/fonc.2019.00390)
Supplement: Supplementary file 2 [file Data_Sheet_1.PDF]

Supplementary Figure Legends

Supplementary Figure 1: Meta-analysis of the pooled HRs of OS for patients divided by p-S6K1 expression status, from Cox multivariate analyses (A). **Supplementary Figure 2:** Meta-analysis of the pooled HRs of OS for esophageal squamous cell carcinoma (A), non-small cell lung cancer (B), nasopharyngeal carcinoma (C), breast cancer (D). **Supplementary Figure 3:** Meta-analysis of the pooled HRs of DFS (A), PFS (B) and RFS (C) for patients with abnormally expressed p-S6K1. **Supplementary Figure 4:** Meta-analysis of the pooled HRs of DFS (A), PFS (B) and RFS (C) for patients with abnormally expressed p-S6.

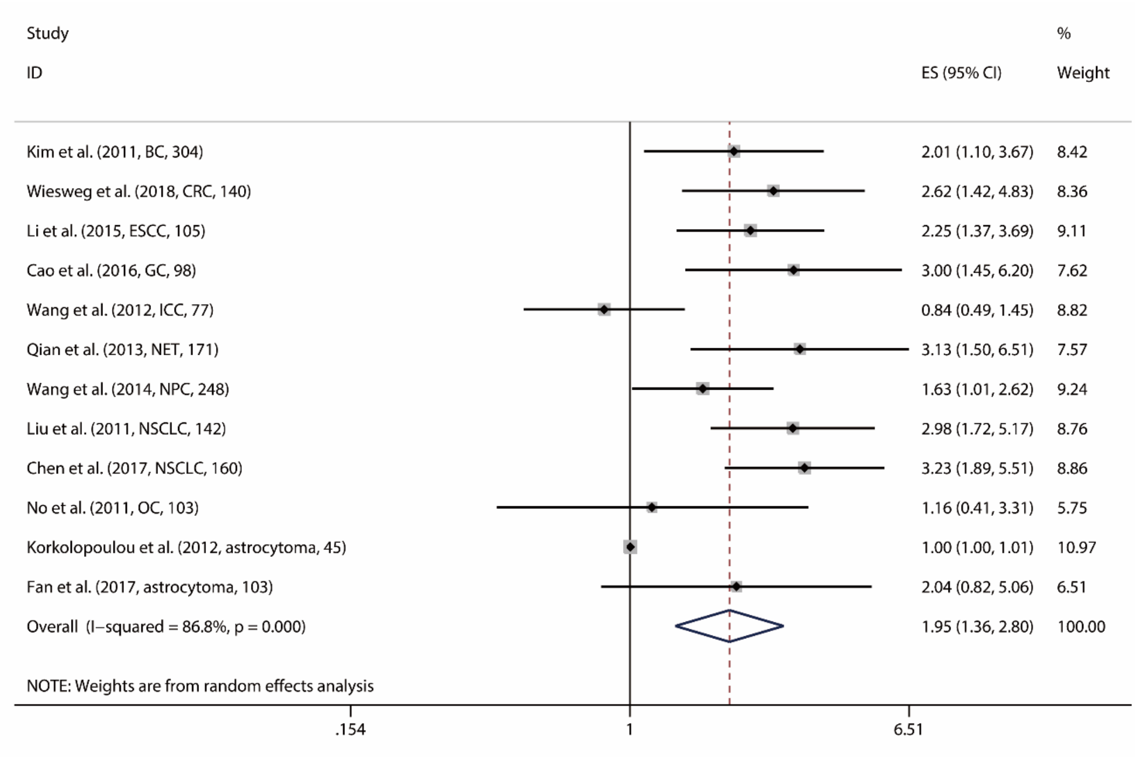

Supplementary Figure 1: Meta-analysis of the pooled HRs of OS for patients divided by p-S6K1 expression status, from Cox multivariate analyses.

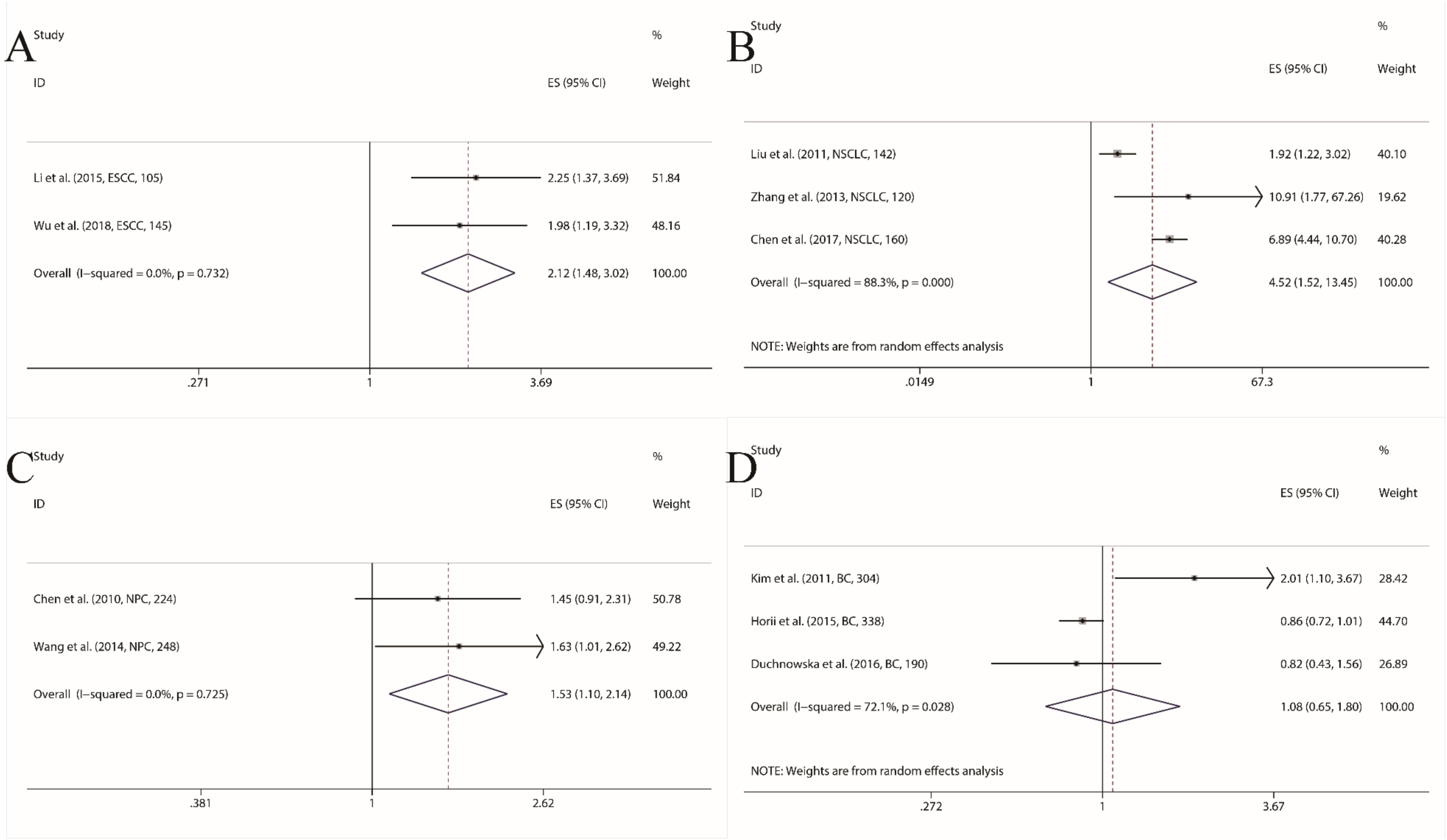

Supplementary Figure 2: Meta-analysis of the pooled HRs of OS for esophageal squamous cell carcinoma (A), non-small cell lung cancer (B), nasopharyngeal carcinoma (C), breast cancer (D).

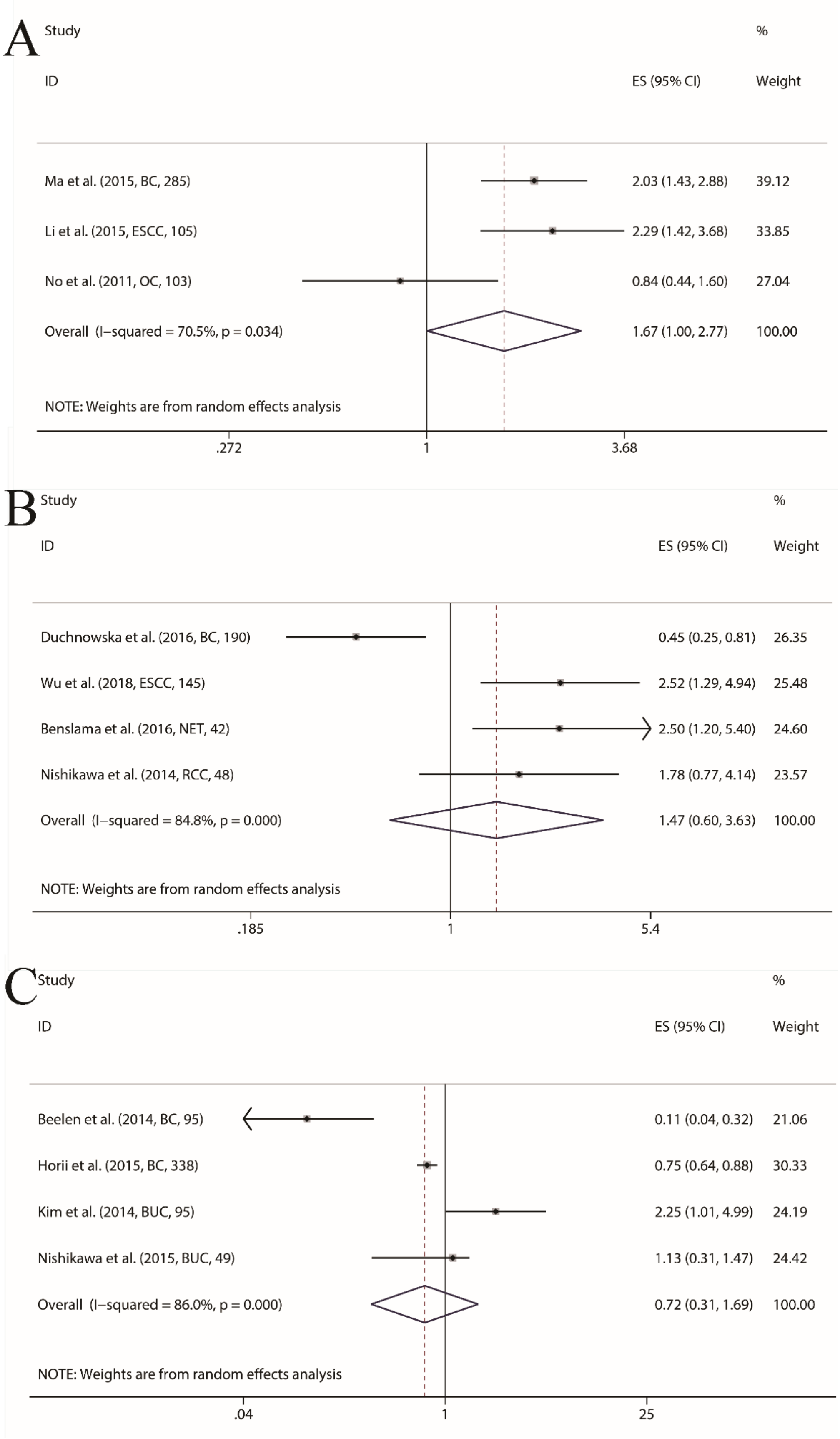

Supplementary Figure 3: Meta-analysis of the pooled HRs of DFS (A), PFS (B) and RFS (C) for patients with abnormally expressed p-S6K1.

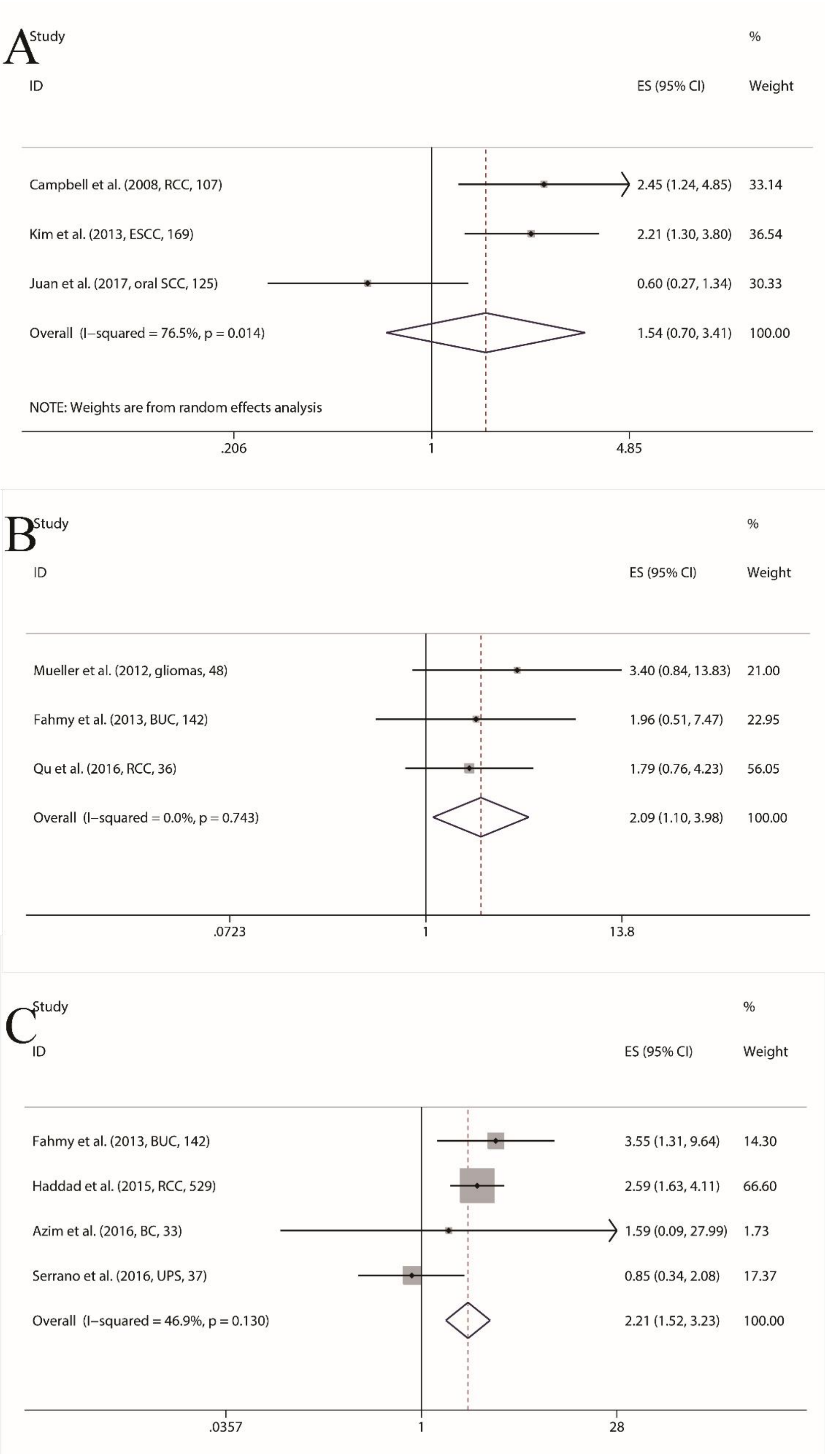

Supplementary Figure 4: Meta-analysis of the pooled HRs of DFS (A), PFS (B) and RFS (C) for patients with abnormally expressed p-S6.
